# Comparative analysis of post-transplant lymphoproliferative disorders after solid organ and hematopoietic stem cell transplantation reveals differences in the tumor microenvironment

**DOI:** 10.1007/s00428-020-02985-4

**Published:** 2020-12-15

**Authors:** Mathis Overkamp, Massimo Granai, Irina Bonzheim, Julia Steinhilber, Jens Schittenhelm, Wolfgang Bethge, Leticia Quintanilla-Martinez, Falko Fend, Birgit Federmann

**Affiliations:** 1 Institute of Pathology and Neuropathology, University Hospital and Comprehensive Cancer Center Tuebingen, Liebermeisterstraße 8, 72076 Tuebingen, Germany; 2 Section of Pathology, Department of Medical Biotechnology, University of Siena, Siena, Italy; 3 Department of Internal Medicine Hematology and Oncology, Comprehensive Cancer Center and University Hospital Tuebingen, Tuebingen, Germany

**Keywords:** Post-transplant lymphoproliferative disease, Solid organ transplantation, Hematopoietic stem cell transplantation, Microenvironment, Macrophages

## Abstract

Post-transplant lymphoproliferative disorders (PTLD) occur after solid organ transplantation (SOT) or hematopoietic stem cell transplantation (HCT) and are frequently associated with Epstein-Barr virus (EBV). Because of the complex immune setup in PTLD patients, the tumor microenvironment (TME) is of particular interest to understand PTLD pathogenesis and elucidate predictive factors and possible treatment options. We present a comparative study of clinicopathological features of 48 PTLD after HCT (*n* = 26) or SOT (*n* = 22), including non-destructive (*n* = 6), polymorphic (*n* = 23), and monomorphic (*n* = 18) PTLD and classic Hodgkin lymphoma (*n* = 1). EBV was positive in 35 cases (73%). A detailed examination of the TME with image analysis-based quantification in 22 cases revealed an inflammatory TME despite underlying immunosuppression and significant differences in its density and composition depending on type of transplant, PTLD subtypes, and EBV status. Tumor-associated macrophages (TAMs) expressing CD163 (*p* = 0.0022) and Mannose (*p* = 0.0016) were enriched in PTLD after HCT. Double stains also showed differences in macrophage polarization, with more frequent M1 polarization after HCT (*p* = 0.0321). Higher counts for TAMs (CD163 (*p* = 0.0008) and cMaf (*p* = 0.0035)) as well as in the T cell compartment (Granzyme B (*p* = 0.0028), CD8 (*p* = 0.01), and for PD-L1 (*p* = 0.0305)) were observed depending on EBV status. In conclusion, despite the presence of immunosuppression, PTLD predominantly contains an inflammatory TME characterized by mostly M1-polarized macrophages and cytotoxic T cells. Status post HCT, EBV positivity, and polymorphic subtype are associated with an actively inflamed TME, indicating a specific response of the immune system. Further studies need to elucidate prognostic significance and potential therapeutic implications of the TME in PTLD.

**Supplementary Information:**

The online version contains supplementary material available at 10.1007/s00428-020-02985-4.

## Introduction

Post-transplant lymphoproliferative disorders (PTLD) are a heterogeneous group of lymphoid or plasmacytic proliferations. They develop in patients under immunosuppression after solid organ transplantation (SOT), or less frequently after allogeneic hematopoietic stem cell transplantation (HCT). PTLDs form a spectrum of usually Epstein-Barr virus (EBV) driven polyclonal proliferations to EBV-positive or EBV-negative clonal malignancies resembling lymphomas occurring in immunocompetent patients. According to the current WHO classification, there are four categories of PTLD [1]: Non-destructive PTLDs show preserved architecture and are usually EBV-positive. Polymorphic PTLDs show significant architectural effacement, are usually EBV positive, and comprise the full range of cellular maturation without fulfilling the criteria for malignant lymphoma. At the end of the spectrum are monomorphic PTLDs which fulfill the criteria for the respective B cell or T/NK-cell lymphomas in immunocompetent patients, and classic Hodgkin lymphoma (CHL). They can be EBV-positive or EBV-negative [1].

PTLD is one of the most serious complications of transplantation with a reported incidence between about 2 and 20% depending on the kind of transplantation and a 3-year survival of about 40 to 55% [2–4]. While the etiology of PTLD is not yet fully understood, the majority of cases, especially early after transplantation, are associated with EBV infection or reactivation, which induces an uncontrolled lymphocyte proliferation [2]. Regarding the etiology of EBV-negative PTLD, hit-and-run EBV infection, the effects of persistent antigen stimulation by the graft, long-term immunosuppression, as well as other infectious agents have been suggested as possible pathogenic mechanisms [2, 5]. Due to advanced conditioning protocols and graft modification, the incidence of EBV-positive PTLD has decreased in recent times resulting in a relative increase of EBV-negative cases [3, 6]. EBV-negative PTLD usually arises late after transplantation and differs in clinicopathological features as well as gene expression profiles from EBV-positive PTLD [3, 5, 7, 8]. This suggests that EBV-negative PTLD might represent a different entity [6, 9] or sporadic lymphoma occurring coincidentally [8].

Adding to its complexity, PTLD can be of donor or host origin. Whereas the vast majority of examined cases of PTLD after HCT is of donor origin [10], PTLD after SOT is usually of host origin [11, 12]. PTLD after HCT is considered to be more aggressive and usually occurs earlier after transplantation [9, 10].

This complex immunologic situation, influenced by the presence of oncogenic EBV, chronic immune stimulation through chronic antigen presentation by the graft, chronic immunosuppression, and interaction of donor-derived immune cells with the host immune cells, makes the tumor microenvironment (TME) of PTLD and interesting focus of research [13], but published data on the TME of PTLD are sparse. The TME represents the specific setting in which a tumor resides and consists of all non-malignant constituents of a neoplasm containing variable numbers of immune cells, mesenchymal cells, blood vessels, and non-cellular components such as extracellular matrix [14]. The composition of the TME has a profound impact on the biological behavior, prognosis, and therapy response in many tumor types including lymphoma, since tumor cells retain a range of dependence on interactions with the non-malignant cells of the TME [13–16].

T cell subsets and tumor-associated macrophages (TAMs) are considered the major immunologically relevant cell types of the TME. TAMs constitute a significant part of the tumor infiltrating microenvironment [14, 17, 18]. They are usually detected using CD163 or CD68 antibodies [19] and further classified corresponding to their functional state as anti-tumoral M1- and pro-tumoral M2-phenotypes in a simplified view [20–22]. In PTLD, the number of TAMs and their polarization appear to correlate with EBV status [23]. Macrophage polarization has been shown to be associated with the T cell composition of tumors [16] as well as prognosis in lymphoma [17, 18, 24, 25].

As comprehensive studies of the specific TME in PTLD are lacking, we aimed to characterize a cohort of PTLD cases after HCT and SOT and focused on differences in TME composition regarding type of transplant, EBV status, and PTLD subtype. In a subset of cases, TME was studied by a detailed digital image-based immunohistochemical analysis with a large panel of antibodies and double stains. Furthermore, EBV status, IGH rearrangement, and PTLD origin (host versus donor) were investigated.

## Material and methods

### Patient selection

Forty-eight patients diagnosed with PTLD at the Institute of Pathology, University Hospital Tuebingen, between 2002 and 2018 were identified. Criteria for inclusion in the study were a confirmed diagnosis of PTLD and documented HCT or SOT. Multiple biopsies have been obtained in seven patients. Except in one case, the biopsy with the first manifestation of PTLD was taken for further studies. Three EBV-negative cases after HCT have been published before [26]. The project was approved by the local Ethics Committee (Tü 096/2016B02).

### Histology and construction of tissue microarray

All cases were reviewed independently by two experienced pathologists (FF and BF) to confirm the diagnosis in accordance with the 2016 revision of the WHO classification of tumors of hematopoietic and lymphoid tissues [1]. Hematoxylin and Eosin (H&E) and Giemsa-stained sections as well as all available immunostains were reviewed. Additional immunostains for completion were performed when necessary. Twenty-two cases with sufficient material were selected for further analyses, and representative tumor areas were marked on H&E slides. The marked tissue areas were used as reference for molecular analyses and for the construction of a tissue microarray (TMA), as described previously [27], using the Manual Tissue Arrayer MTA-Booster-01 (Beecher Instruments Inc.). Three cores of 0.6 mm in diameter were taken per case.

### Immunohistochemistry

Extended immunohistochemical analyses including antibodies against CD15, CD20, CD30, PAX5, MUM1, P53, MYC, Kappa, Lambda, CD3, CD4, CD5, CD8, and CD56; TIA1, FOXP3, Granzyme B, FOXP1, PD1, and PD-L1; as well as the macrophage markers CD68, CD163, cMaf, Mannose, and pStat1 were performed using serial sections from the TMA (detailed information on the antibodies used can be found in the *Supplementary Table* *S1**).* IHC staining was performed using an automated immunostainer (Ventana Medical Systems, Tucson, AZ, USA), according to the manufacturer’s protocol. Additionally, CD163/pStat1 and CD163/cMaf double stains were performed to detect M1- or M2-polarization of macrophages, respectively [22]. For the CD163/pStat1 double stain, the pStat1 antibody was used as first primary antibody, and the detection of the bound antibodies was performed using ULTRA Red detection kit. For the CD163/cMaf double stain, the cMaf antibody was used as first antibody, and the detection of the bound antibody was performed using OptiView DAB detection kit. The CD163 antibody was incubated in both cases posteriorly, followed by detection with OptiView DAB detection kit or ULTRA Red detection kit, respectively (detailed information on the antibodies used can be found in the *Supplementary Table* *S1*) The absolute numbers of CD163/pStat1-positive and CD163/cMaf-positive cells were evaluated independently by two experienced pathologists (BF and MG).

### EBV detection and latency type

The presence of EBV infection was determined in all cases using in situ *hybridization* for Epstein-Barr encoding region (EBER-ISH) according to the manufacturer’s protocol (Ventana Medical Systems, Tucson, AZ, USA). EBV latency was determined using staining for latent membrane protein 1 (LMP1) and EBV nuclear antigen 2 (EBNA2) latency proteins. Cases were classified as latency type I (EBER+, LMP1−, EBNA2−), latency type II (LMP1+, EBNA2−), or latency type III (LMP1+, EBNA2+).

### Digital image analysis and automated quantification

All TMA slides were digitalized using Zeiss Mirax Scanner (Carl Zeiss Microscopy GmbH, Jena, Germany) or Roche Ventana DP 200 Slide Scanner (Ventana Medical Systems Inc., Tucson, AZ, USA). High-resolution digital MIRAX- or TIFF files with × 20 magnification were created for digital image analyses. For precise quantitative analysis of immunohistochemical stains, all TMA slides were analyzed using Definiens Tissue Studio (Version 4.3., Definiens AG, Munich, Germany). Using automatic tissue detection, each core was identified by the software. For each core, a region of interest (ROI) was detected either automatically or manually, excluding artifacts and non-representative tissue areas.

For the detection of positive cells, Tissue Studio was calibrated individually for each IHC stain to produce the best possible results, using refined versions of the software’s predefined solutions. Tissue Studio was calibrated to detect the number of all cells in each core, the number of IHC-negative cells in each core, and the number of IHC-positive cells in each core. This data was exported, and the percentages of IHC-positive cells among all cells were calculated for each individual core. For each marker, the arithmetic mean of the three cores corresponding to one case was calculated and used for further analyses. The results of Definiens Tissue Studio analyses were validated visually for each individual core. If Definiens Tissue Studio analysis failed due to compromised staining or compromised tissue, the percentages of positive cells were determined visually by experienced pathologists.

### DNA isolation, clonality analysis, and microsatellite instability analysis

Genomic DNA was extracted from macrodissected 5-μm paraffin sections using the Maxwell® RSC DNA FFPE Kit and the Maxwell® RSC Instrument (Promega, Madison, WI, USA) according to the manufacturer’s instructions. The PCR for IGH gene rearrangements (FR1-FR3) a kappa VJ and kde rearrangements were performed in accordance with the BIOMED-2 guidelines as previously described [28–30]. The JH primer was modified with D4 fluorescent dyes (Sigma-Aldrich, St. Louis, MO, USA). For GeneScan analysis, 0.5 μl of the PCR products were mixed with sample loading solution containing 0.24 μl DNA Size Standard 400 (Beckman Coulter, Brea, CA, USA). For detecting donor or host origin of PTLD, microsatellite instability analysis (MSI) of PTLD tissue and normal tissue of non-hematological origin that had been retrieved before PTLD diagnosis was performed, as previously described [31]. More precisely multiplex PCR for BAT25 + 26, D2S123, D5S346, and D17S250 markers and analyses of the fragment sizes of the PCR products were performed. For clonality and MSI analysis, the PCR products were separated by capillary electrophoresis using the GenomeLab GeXP Genetic Analysis System (Beckman Coulter, Carlsbad, CA, USA). For GeneScan analysis, 0.5 μl of the PCR products were mixed with sample loading solution containing 0.24 μl DNA Size Standard 400 (Beckman Coulter, Brea, CA, USA).

### Statistical analysis

To compare the quantitative data, Student’s *t* test for independent variables was used for comparing continuous variables and Pearson’s chi-square test for categorical variables. Statistical significance was concluded for values of *p* < 0.05. Data was analyzed using JMP® (Version 15.1.0. SAS Institute Inc., Cary, NC, 1989-2020, SAS Institute Inc.).

## Results

### Clinical and morphological features

A summary of the clinical features of all cases included in this study is shown in Table 1. Patients (13 females/35 males) had a median age of 31 years (range 1–74 years) at diagnosis of PTLD. PTLD occurred after HCT in 26 cases and after SOT in 22 cases. The median interval from transplantation to the first manifestation of PTLD was 13 months (range 1–435 months). Cases after HCT occurred after a median of four months (range 1–228 months), while cases after SOT occurred later after a median of 47 months (range 3–435 months, *p* = 0.0146). There were 35 EBV-positive cases, which occurred after a median of 6 months (range 1–435 months), and 13 EBV-negative cases after a median of 75 months (range 3–217 months; *p* = 0.0295). After HCT there were five cases of EBV-negative (5/26, 19%) in contrast to eight EBV-negative cases after SOT (8/22, 36%). *Supplementary Table* *S2* shows the clinicopathological data of the total collective on a case by case basis.

**Table 1:** Characteristics of the total collective

Diagnosis	*n* = patients	Age in years at diagnosis of PTLD median (range)	Sex		Tx		EBV		Months Tx to PTLD median (range)	Follow-up		
			f	m	HCT	SOT	+	-		Alive	Dead	n/a
Total	48	31 (1–74)	13	35	26	22	35	13	13 (1–435)	28	15	5
Non-destructive PTLD	6	19 (5–74)	2	4	3	3	5	1	23 (2–201)	4	2	0
Polymorphic PTLD	23	38 (1–71)	5	18	15	8	22	1	5 (1–435)	12	8	3
Monomorphic PTLD	18	33 (4–69)	5	13	7	11	7	11	48 (2–217)	11	5	2
Classic Hodgkin lymphoma PTLD	1	19	1	0	1	0	1	0	228	1	0	0

*PTLD* post-transplant lymphoproliferative disorder, *EBV* Epstein-Barr virus, *Tx* transplantation, *m* male, *HCT* hematopoietic stem cell transplantation, *f* female, *SOT* solid organ transplantation, *n/a* not available

**Fig. 1:**
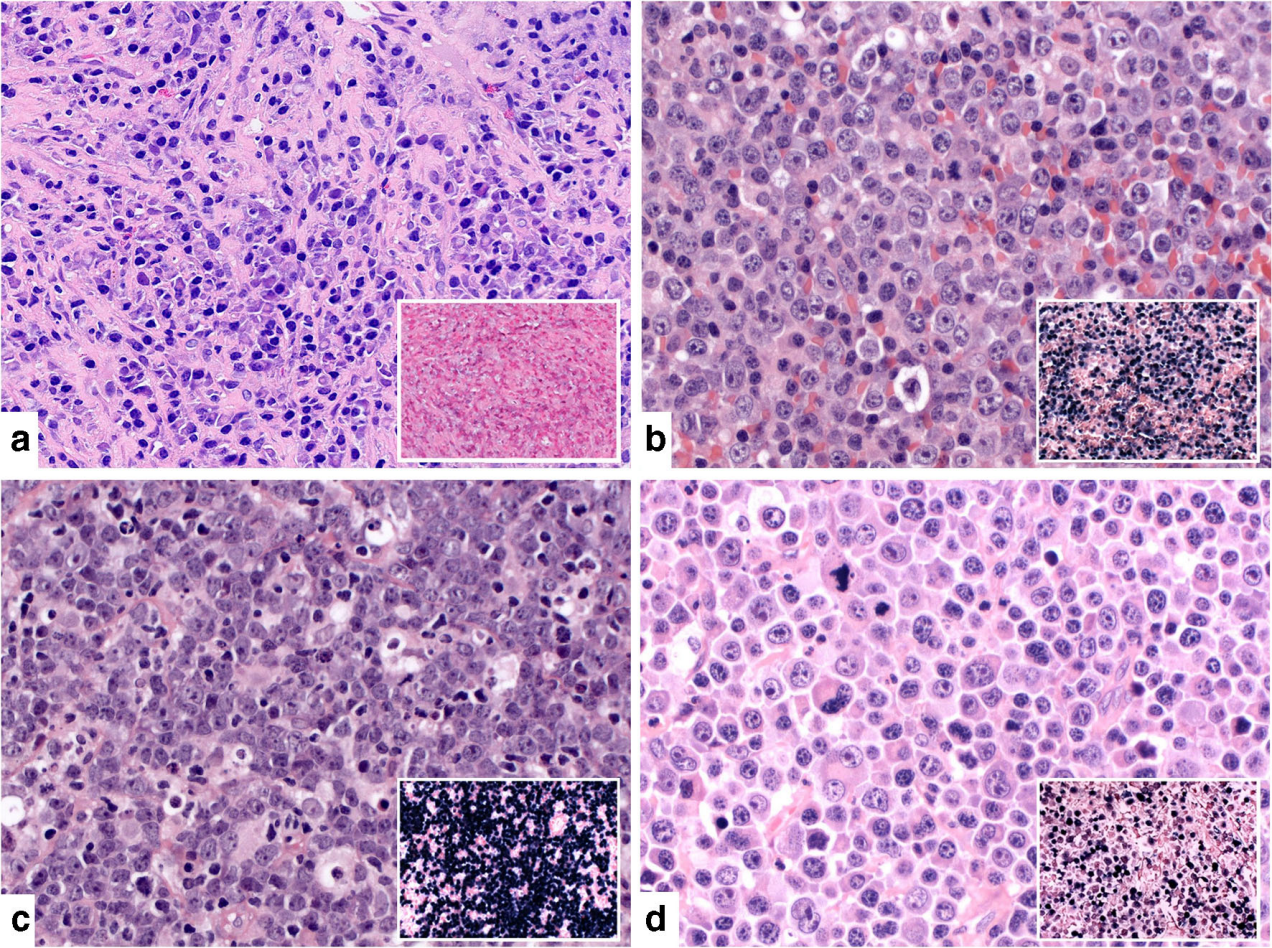
Morphological features of four different PTLD cases. **a** Non-destructive PTLD in the liver, EBV negative (case # 24). The infiltrate is composed of CD138-positive plasma cells with polytypic light chain expression (data not shown) and fibrosis. Hematoxylin and Eosin stain (H&E), original magnification × 400; insert EBER in situ hybridization with few positive cells, × 100). **b** Polymorphic PTLD, EBV positive (case # 2). The case shows the polymorphic spectrum of a lymphoid proliferation with immunoblasts, plasma cells, and small lymphocytes (H&E, × 400; insert EBER in situ hybridization, × 100). **c** Monomorphic PTLD, Burkitt lymphoma, EBV positive (case # 17). The infiltrate consists of medium-sized, monomorphic tumor cells with basophilic cytoplasm and starry sky macrophages. The tumor cells were positive for CD20, CD10, BCL-6, and MYC with a corresponding t(8;14) translocation detected by FISH (data not shown) (H&E, × 400; insert EBER in situ hybridization, original magnification × 100). **d** Monomorphic PTLD, plasmablastic lymphoma, EBV positive (case # 21). The infiltrate is composed of large tumor cells exhibiting plasmablastic features with large nuclei and prominent nucleoli and intermingled multinucleated cells. The cells were positive for MUM1 and CD138 and negative for CD20 and showed kappa light chain restriction (data not shown) (H&E, × 400; insert EBER in situ hybridization, × 100)

Reclassification of all cases revealed six cases of non-destructive PTLD (12.5%), including one unusual EBV-negative case with plasmacytic hyperplasia in the liver (Fig. 1a), 23 polymorphic PTLD (48%) (Fig. 1b), 18 monomorphic PTLD (37.5%) (Fig. 1c and d), and one case of CHL PTLD (2%). For further analyses, the case of CHL PTLD was subsumed under monomorphic PTLDs. The median interval between transplantation and diagnosis of the first manifestation of PTLD was lowest in polymorphic (5 months, range 1–435 months) after exclusion of an unusual outlier, an EBV-positive polymorphic PTLD arising 36 years after kidney transplantation, and highest in monomorphic PTLD (60 months, range 2–218 months, *p* = 0.001). The ratio of polymorphic versus monomorphic cases was slightly higher after HCT (15/11) versus SOT (11/11).

Follow-up data were available in 43/48 patients. At the time of last follow-up, 28 patients were alive (4/6 with non-destructive PTLD, 12/23 with polymorphic PTLD, and 12/19 with monomorphic PTLD), while 15 patients were dead.

### EBV status, IGH clonality, and microsatellite analyses

A subgroup of 22 cases (19 males/3 females) was selected for TMA construction and a detailed immunophenotypic and molecular analysis based on tissue availability. The patient characteristics and PTLD features were representative for the entire collective (*Supplementary Figure* *S1*) and are shown for each TMA case in detail in Table 2.

**Table 2:** Clinical and histological data of the TMA cases

TMA #	Age (years)	Sex	Diagnosis	Subtype	EBV	Latency	Tx	Underlying disease	Biopsy site	Months Tx to PTLD	Follow-up
*1*	9	m	Non-destructive PTLD	FFH	+	I	HCT	cALL	LN	22	Alive
*2*	36	m	Polymorphic PTLD		+	III	HCT	T-ALL	LN	5	n/a
*3*	52	m	Polymorphic PTLD		+	III	HCT	AML	LN	2	Alive
*4*	28	m	Polymorphic PTLD		+	III	HCT	AML	LN	2	Dead, died of PTLD
*5*	52	m	Polymorphic PTLD		+	III	HCT	AML	LN	4	Alive
*6*	17	m	Polymorphic PTLD		+	I	HCT	NLPHL	LN	2	Dead, died of underlying disease
*7*	15	m	Polymorphic PTLD		-	NA	HCT	T-ALL	LN	3	Alive
*8*	52	m	Polymorphic PTLD		+	III	HCT	Follicular lymphoma	Tonsil	19	Alive
*9*	46	m	Monomorphic PTLD	DLBCL	+	III	HCT	AML	Thyroid	23	Alive
*10*	28	m	Monomorphic PTLD	PBL	-	NA	HCT	cALL	Muscle/ soft tissue	75	Alive
*11*	29	m	Monomorphic PTLD	PBL	+	III	HCT	AML	LN	2	Dead, died of PTLD
*12*	19	f	Classic Hodgkin Lymphoma PTLD		+	II	HCT	Hereditary malignant osteopetrosis	LN	228	Alive
*13*	32	m	Non-destructive PTLD	PH	+	I	Liver	Cryptogenic liver failure	LN	201	Alive
*14*	20	m	Non-destructive PTLD	FFH	+	I	Liver	Acute liver failure	LN	12	Alive
*15*	66	m	Polymorphic PTLD		+	I	Liver	HCC	Liver	6	Alive
*16*	4	f	Monomorphic PTLD	DLBCL	+	III	Intestine	Intestinal aganglionosis	Tonsil	8	Alive
*17*	13	m	Monomorphic PTLD	BL	+	I	Liver	Biliary atresia	LN	36	Alive
*18*	10	m	Monomorphic PTLD	DLBCL	-	NA	Kidney	Obstructive uropathy	LN	115	Alive
*19*	36	m	Monomorphic PTLD	DLBCL	-	NA	Kidney	IgA nephropathy	Mesentery	86	Alive
*20*	21	m	Monomorphic PTLD	DLBCL	-	NA	Kidney	Diffuse mesangial sclerosis	Small intestine	217	Alive
*21*	40	f	Monomorphic PTLD	PBL	+	III	Kidney	n/a	Small intestine	n/a	n/a
*22*	11	m	Monomorphic PTLD	DLBCL	-	NA	Liver	Biliary atresia	LN	84	Alive

*PTLD* post-transplant lymphoproliferative disorder, *PH* plasmacytic hyperplasia, *cALL* common acute lymphoblastic leukemia, *Tx* Transplantation, *FFH* florid follicular hyperplasia, *T-ALL* T cell acute lymphoblastic leukemia, *HCT* hematopoietic stem cell transplantation, *DLBCL* diffuse large B cell lymphoma, *AML* acute myeloid leukemia, *EBV* Epstein-Barr virus, *PBL* Plasmablastic lymphoma, *NLPHL* nodular lymphocyte predominant Hodgkin lymphoma, *m* male, *BL* Burkitt lymphoma, *HCC* hepatocellular carcinoma, *f* female, *LN* lymph node, *n/a* not available, *TMA* tissue microarray, *NA* not applicable

**Fig. 2:**
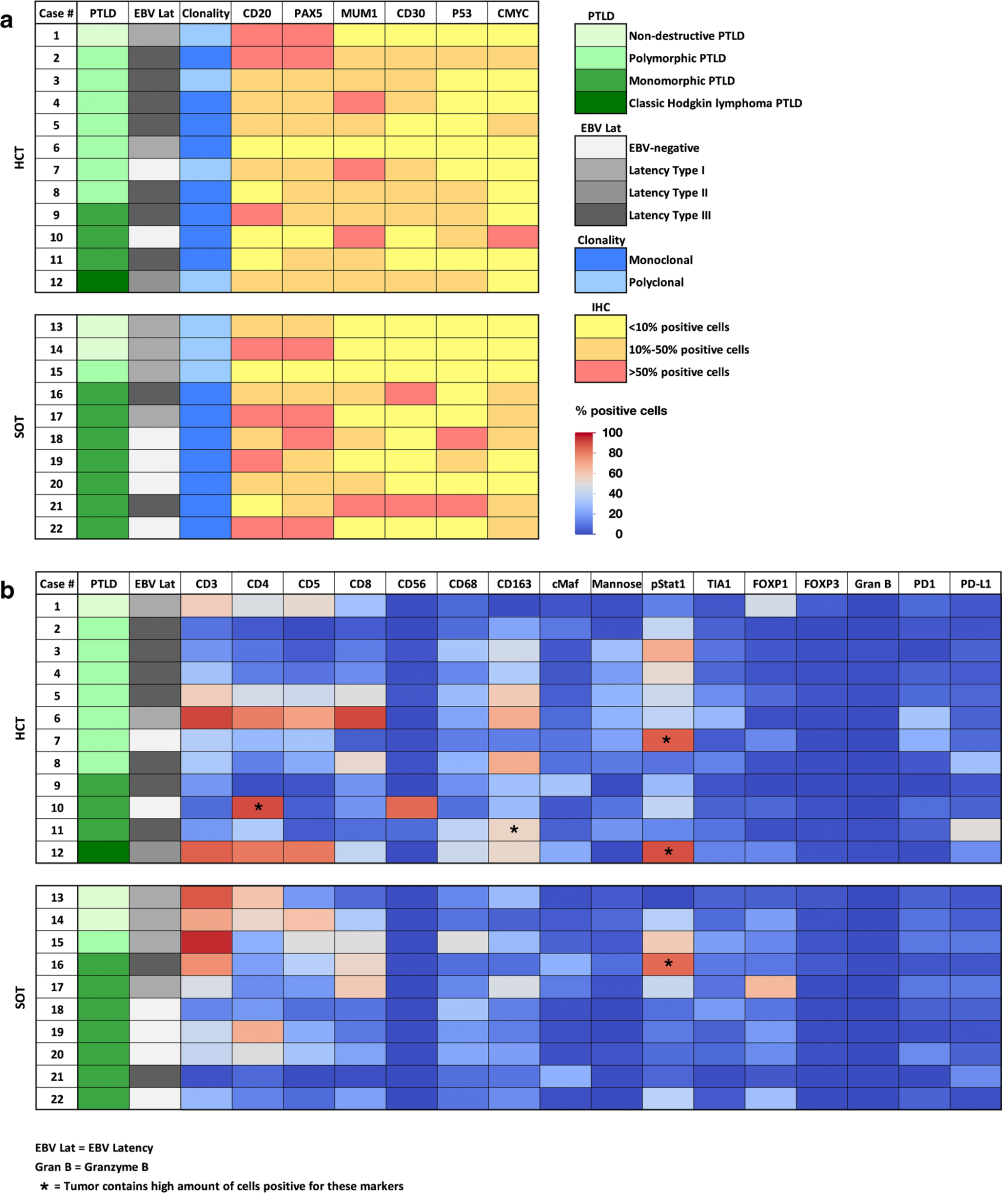
Pathological findings and immunohistochemical analysis of the PTLD microenvironment of the 22 cases analyzed on the tissue microarray. **a** The cases are grouped according to the transplant status and subgrouped based on PTLD diagnosis group. B cell clonality analyses detected monoclonality with immunoglobulin heavy chain (IgH)-rearrangement in 15 cases. In case #19, monoclonality was only apparent in an analysis of the immunoglobulin kappa light chains. The EBV status and the latency type as well as specific B cell marker and the p53 status are also shown. **b** The heatmap shows the percentages of positive cells for each individual marker are listed. Note the high percentages of positive cells in the T cell compartment as well as in the macrophage markers CD163 and pStat1

Figure 2 summarizes immunophenotypical findings. The three cases of non-destructive PTLD analyzed were EBV-positive (type I latency) (Fig. 2). All but one case of polymorphic PTLD were EBV-positive. Only one case of polymorphic PTLD was EBV-negative (case #7, *Supplementary Figure* *S2*). EBV was present in 5/11 cases of monomorphic PTLD, all latency 3 except a case of Burkitt lymphoma, expressing latency I (Fig. 2). IGH clonality analysis revealed polyclonality in all cases of non-destructive PTLD and in three cases of polymorphic PTLD, while five cases were monoclonal. All cases of monomorphic PTLD were monoclonal.

Comparative microsatellite analysis demonstrated donor origin of all PTLD after HCT (10/10). Three of four examined cases after SOT were of host origin. Of interest, one case after SOT with PTLD manifestation in the transplanted liver was of donor origin.

### Tumor microenvironment

A detailed study of the TME was performed on the TMA containing triplicates of the 22 cases (Table 2). For the comparative analyses, cases of non-destructive PTLD were excluded. The antibody panel was aimed at characterizing the reactive immune cell infiltrate and at highlighting potential differences in the TME of distinct PTLD subgroups. The results of the immunohistochemical analysis of the specific tumor microenvironment are represented in Fig. 2b on a case by case basis. The heatmap shows an enrichment of tumor-associated macrophages (TAMs), as well as T cells subsets implicating an immune cell-rich environment.

TME was analyzed according to type of transplant, EBV status, and diagnostic categories of PTLD. The data are summarized in Table 2. PTLD after HCT showed an enrichment of macrophages expressing CD163 (*p* = 0.0022) and Mannose (*p* = 0.0016) and activated cytotoxic cells positive for Granzyme B (*p* = 0.0282) compared to cases after SOT, with less cells positive for FOXP1 (*p* = 0.027). Similarly, EBV-positive cases showed significantly higher numbers of CD163 (*p* = 0.0008) and cMaf (*p* = 0.0035)-positive macrophages, as well as more CD8+ T cells (*p* = 0.01) expressing Granzyme B (*p* = 0.0028) compared to EBV-negative cases. Furthermore, PD-L1 expression of the total infiltrate including tumor cells was increased in EBV-positive cases (*p* = 0.0305). Polymorphic PTLD contained more macrophages positive for CD163 (*p* = 0.606), Mannose (*p* = 0.0049), and pStat1 (*p* = 0.0973). pStat1 was used as a marker for M1 polarization and Mannose and cMaf as markers for M2 polarization of the macrophages.

**Fig. 3:**
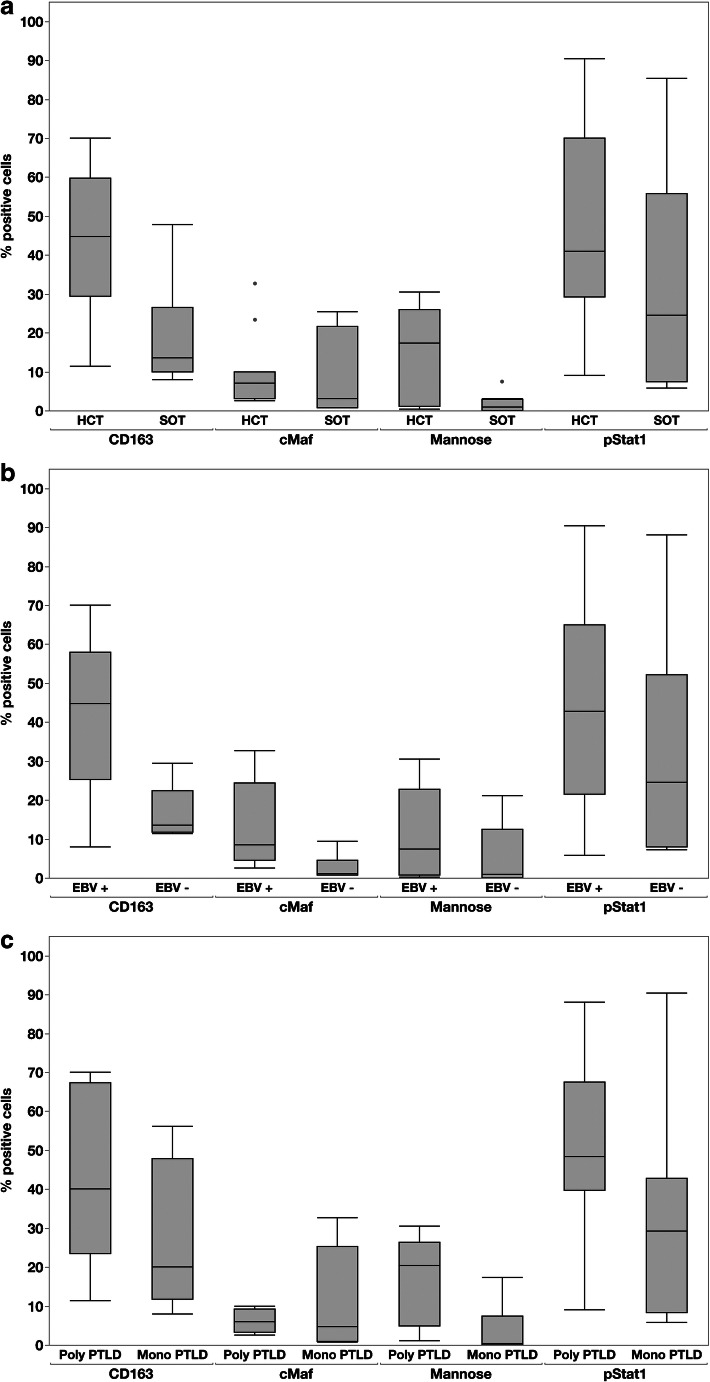
Statistical analysis of the PTLD microenvironment. The Box plots show the association of transplant status, EBV status, and PTLD diagnosis group with different marker capturing the microenvironment like CD163 as well as cMaf, pStat1, and Mannose to define the polarization status of the macrophages. pStat1 would indicate M1 polarization, whereas cMaf and Mannose would suggest M2 polarization. **a** Hematopoietic stem cell transplantation versus (vs.) solid organ transplantation: A significant association is shown for CD163 (*p* = 0.0022) and Mannose (*p* = 0.0016). **b** EBV-positive cases vs. EBV-negative cases: A significant association is shown for CD163 (*p* = 0.0008) and cMaf (*p* = 0.0035). **c** Polymorphic (Poly) vs. Monomorphic (Mono) PTLD: A significant association is shown for Mannose (*p* = 0.0049). All analyzed with two-sample *t* test

**Fig. 4:**
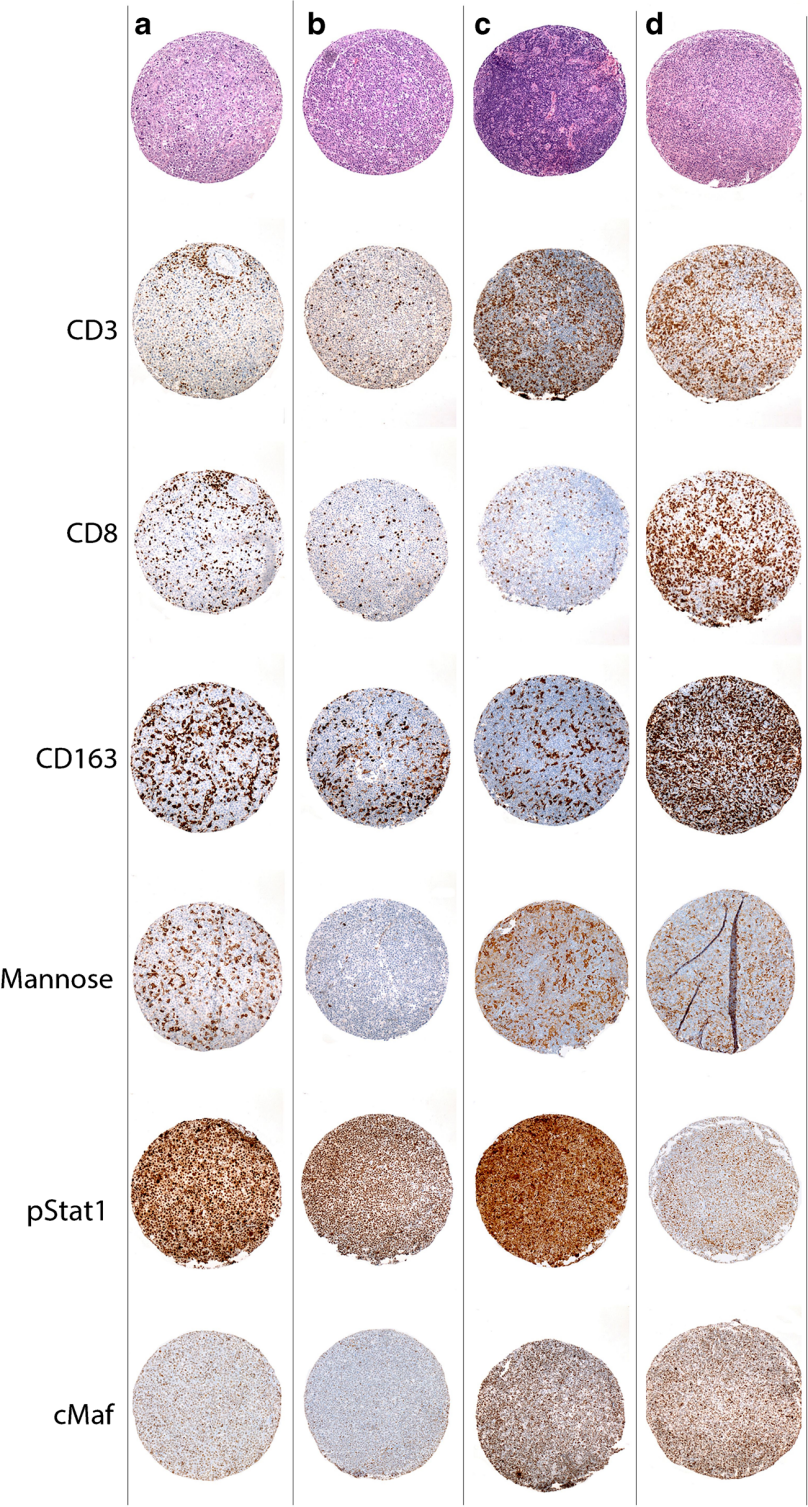
Different immunostains for the microenvironment in PTLD in four exemplary cases. In **a** and **b,** the increased expression of CD163 and Mannose in PTLD after hematopoietic stem cell transplantation (HCT) compared with PTLD after solid organ transplantation (SOT) is shown (*p* = 0.0022 and 0.0016, respectively). (**a**, case # 10, HCT monomorphic PTLD; **b**, case # 18, SOT monomorphic PTLD; both, original magnification × 100). In **c** and **d**, the association of EBV status with an increase in CD163+ macrophages, CD8+ T cells, and cMaf (*p* = 0.0008, 0.01 and 0.0035, respectively) is demonstrated (**c**, case # 7, EBV-negative polymorphic PTLD; **d**, case #8, EBV-positive polymorphic PTLD; both, original magnification × 100)

Comparisons for selected antibodies are shown in Fig. 3a–c and illustrated in Fig. 4.

In order to exclude that the differences between SOT and HCT cases were due to interdependence of variables, we looked only at the subgroup of EBV-positive cases (HCT versus SOT: CD163 *p* = 0.0316, Mannose *p* = 0.0117), as well as at the monomorphic cases (HCT versus SOT: CD163 *p* = 0.0156), confirming significant differences in macrophage content.

**Fig. 5:**
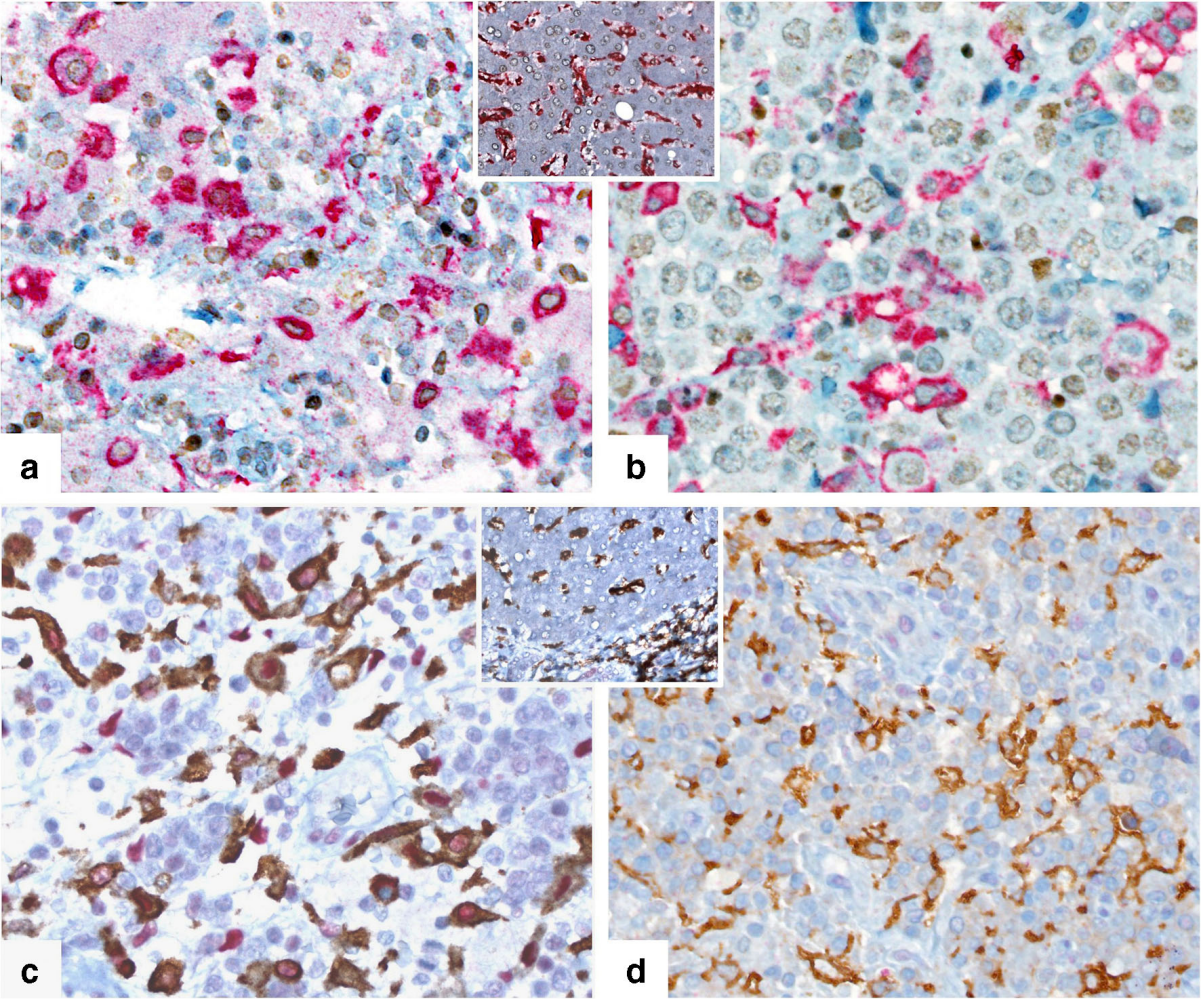
Macrophage polarization. The double stains identify the polarization status of the macrophages. This figure highlights the different staining pattern in four exemplary cases. **a** Case # 13, M2-polarized PTLD, CD163+/cMaf+ stain; **b** case # 10, M1-polarized PTLD, CD163+/cMaf-stain; insert in **a**–**b**, CD163/cMaf, liver tissue control (**a**–**b**, insert: CD163 red membranous, cMaf brown nuclear; all original magnification × 400). **c** Case # 20, M1-polarized PTLD, CD163+/pStat1+. An interesting observation is the positivity of some tumor cells in the staining for pStat1. **d** Case # 13, M2-polarized PTLD, CD163+/pStat1-; insert in **c**–**d**, CD163/pStat1, liver tissue control (**c**–**d**, insert: CD163 brown membranous, pSTAT1 red nuclear; all original magnification × 400)

To further explore the differences detected in macrophage subpopulations, double stains with CD163/pStat1 and CD163/cMaf were performed, representing M1 and M2 polarization, respectively (Fig. 5). To determine the polarization status, we calculated the ratio of CD163/pStat1-positive cells to CD163/cMaf-positive cells for each case (M1, ratio of CD163/pStat1-+ cells: CD163/cMaf+ cells > 1.5; M2, ratio of CD163/cMaf+ cells: CD163/pStat1+ cells > 1.5; no polarization (intermediate), neither ratio > 1.5) [22]. *Supplementary Table* *S3* shows the polarization status for each of the 22 cases. Notably, 10 cases in the HCT group were classified as M1-polarized representing a pro-inflammatory immune status, while two cases did not exhibit polarization. In contrast, six cases were classified in the SOT group as M1 polarized and four cases as M2 polarized (*p* = 0.0321). The comparison regarding EBV status and PTLD subtype did not show statistically significant differences.

## Discussion

In this study, we compared the clinicopathological features of 48 cases of PTLD after either HCT or SOT, with a detailed analysis of the tumor-specific microenvironment by digital image-based quantitative immunohistochemistry in a subset of patients. Despite the presence of immunosuppression, PTLDs are characterized by an immune cell-rich, inflammatory TME. Type of transplant, EBV status, and PTLD subtype are major factors influencing TME composition, especially regarding T cell subsets and the number and polarization of CD163-positive macrophages.

The composition and clinicopathological features of our PTLD collective are comparable to published data, although we had a relatively high percentage of polymorphic PTLD with a total of 23/48 cases (48% vs 19% [32]/28% [33]) [4]. Important parameters determining the classification of PTLD are EBV status and the specific immunodeficiency setting [3, 5, 6, 24]. Although the percentage of EBV-negative PTLD after SOT has increased in recent years, the relatively high number of EBV-negative cases after HCT (5/26) is surprising [34] including two unusual EBV-negative cases, a polymorphic PTLD occurring 3 months after HCT and an EBV-negative non-destructive PTLD in the form of plasmacytic hyperplasia occurring 23 months after HCT [6]. Possibly due to the longer follow-up in our series, we observed more EBV-negative cases than reported by others, including three cases of this series published previously [26]. With significant differences in the intervals between transplantation and PTLD dependent on EBV status (*p* = 0.0295), type of transplant (*p* = 0.0146) [34], and PTLD subtype, a bimodal distribution could be confirmed for these clinical parameters [9, 35]. In agreement with published data, all tested PTLD after HCT arose from donor lymphocytes [36] except one case of EBV-positive polymorphic PTLD of donor origin after liver transplantation with the PTLD manifesting in the transplanted organ [11, 37, 38].

The composition of the tumor microenvironment plays a pivotal role in the pathogenesis of tumors and has been shown to be of prognostic importance [14, 24, 39, 40]. Due to the unique immunologic setup in PTLD patients, the characterization of the TME is of great interest and may have therapeutic implications. TME in PTLD is impacted through a complex interplay between chronic antigenic stimulation caused by the graft organ, immunosuppressive therapy, EBV infection, and the interaction with donor-derived immune cells accompanying the graft [13]. After HCT, the reconstitution process that the transplanted immune system has to undergo adds additional influences. Under the expectation that differences in the TME between different subgroups of PTLD might be subtle and of quantitative nature, digital image analysis in order to obtain a more objective TME assessment was used [41]. With this approach, the presence of an immune cell-rich TME with an enrichment of CD163-positive macrophages was demonstrated, despite the presence of immunosuppression [39]. A detailed analysis of macrophages is especially important when studying the TME since they have the capacity to exert both pro- and antitumor activity [20]. At first glance, our results imply an immunosuppressive environment, since CD163 is considered a marker for M2 macrophages [20, 21]. This simplistic approach is now being questioned, since CD163-positive macrophages can also express M1-specific markers [22]. To establish the ratio between M1 and M2 polarization, double stains for CD163/pStat1 as a marker for M1-polarization and CD163/cMaf as markers for M2-polarization were performed [21, 22]. This approach demonstrated a predominance of M1-polarization, which was more pronounced in PTLD after HCT versus SOT (*p* = 0.0321), possibly reflecting the immune reconstitution after HCT.

In addition to differences in polarization of macrophages, their number and phenotype varied depending on type of transplant, EBV status, and type of PTLD. An increase of CD163-positive macrophages was present after HCT (*p* = 0.0022), in EBV-positive cases (*p* = 0.008) and in polymorphic compared to monomorphic PTLD, confirming previous reports [23, 42]. Another hint towards a more inflammatory background in cases after HCT was the reduced expression of FOXP1 (*p* = 0.027), which is described as negative regulator of immune response [43] and is overexpressed in EBV-negative PTLD [7].

Considering the impact of EBV in the pathogenesis of PTLD and the role of cytotoxic T cells in antiviral response, not surprisingly, we found increased numbers of CD8-positive T cells (*p* = 0.01) and Granzyme B-positive cytotoxic effector cells (*p* = 0.0028), which can also include TAMs [2, 13], indicating a more cytotoxic environment [15, 42, 44].

PD-L1 and PD1 play an important role in the immune evasion by tumor cells and, therefore, are of interest in the setting of lymphoproliferations arising in a background of immunosuppression [45]. In PTLD, the role of PD-L1 is still controversial, with some studies failing to detect a correlation of PD-L1 expression with EBV-status [46] and others reporting high PD-L1 expression in EBV-positive cases in agreement with our findings [47].

Taken together, our data suggest that PTLD after HCT corresponds to an immunologically “hot tumor” setting in which the transplanted immune system, being in a state of regeneration, initiates an anti-viral and thus anti-PTLD reaction. PTLD after SOT, in contrast frequently occurs later and the environment is affected by a long-lasting iatrogenic immunosuppression more commonly resulting in an immunosuppressive TME, especially in the monomorphic subtype. Of interest, 2/4 cases of monomorphic PTLD with M2 polarization showed a strong expression of p53 indicative of *TP53* mutation, in accordance with mouse model data demonstrating that the loss of p53 initiates a polarization of macrophages towards M2 [48].

Although this retrospective study represents the first comprehensive description of the TME in PTLD with special emphasis on the type of transplant, our work has some limitations. The small number of cases makes a robust analysis of subgroups difficult and does not allow considering other potentially confounding clinical factors such as the type of immunosuppression or presence of GVHD, which might have major impact on PTLD development and evolution. Therefore, larger sample sizes are required in future studies to enable matched pair analyses and direct correlation with outcome.

In summary, our comparative analysis shows the broad clinicopathological spectrum of PTLD after HCT and SOT and demonstrates the presence of a predominantly inflammatory TME significantly influenced by the type of transplant, EBV status, and PTLD subtype, reflecting the complexity of the immune response.

## Supplementary information

ESM 1 (PDF 1680 kb)

## Data Availability

All data generated or analyzed during this study are included in this published article and its supplementary information files
