# Supplementary material for: Comparative analysis of post-transplant lymphoproliferative disorders after solid organ and hematopoietic stem cell transplantation reveals differences in the tumor microenvironment
Source: Virchows Arch. 2020 Dec 15;478(6):1135–48. doi: 10.1007/s00428-020-02985-4 (PMC8203555; doi:10.1007/s00428-020-02985-4)

Supplementary Information
Overkamp et al.

Supplementary Table S1……………………………………………………………………………………………………..2

Supplementary Table S2……………………………………………………………………………………………………..3

Supplementary Table S3……………………………………………………………………………………………………..5

Supplementary Figure S1……………………………………………………………………………………………………6

Supplementary Figure S2……………………………………………………………………………………………………7

Supplementary Table S1 *List of the antibodies*

**Marker**	**Clone**	**Dilution**	**Company**
**CD3**	2FGV6	Ready to use	Roche (Basel, Switzerland)
**CD4**	SP35	1:50	Zytomed (Berlin, Germany)
**CD5**	SP19	1:100	Medac (Wedel, Germany)
**CD8**	C8/144B	1:300	Dako (Santa Clara, CA, United States)
**CD15**	MMA/LeuM1	1:100	Biozol (Eching, Germany)
**CD20**	L26	1:400	Dako (Santa Clara, CA, United States)
**CD30**	Ber-H2	1:30	Dako (Santa Clara, CA, United States)
**CD56**	MRQ-24	1:300	Menarini (Florence, Italy)
**CD68**	KP1	1:5000	Dako (Santa Clara, CA, United States)
**CD163**	MRQ-26	1:150	Medac (Wedel, Germany)
**cMaf**	BLR045F	1:750	Abcam (Cambridge, United Kingdom)
**cMyc**	y69	Ready to use	Roche (Basel, Switzerland)
**EBNA2**	PE2	1:1000	Abcam (Cambridge, United Kingdom)
**FOXP1**	SP133	1:100	Abcam (Cambridge, United Kingdom)
**FOXP3**	236A/E7	1:50	Abcam (Cambridge, United Kingdom)
**Granzyme B**	poly Rabbit	Ready to use	Roche (Basel, Switzerland)
**Kappa**	poly Rabbit	1:25000	Dako (Santa Clara, CA, United States)
**Lambda**	poly Rabbit	1:25000	Dako (Santa Clara, CA, United States)
**LMP**	CS 1-4	1:400	Medac (Wedel, Germany)
**Mannose**	Polyclonal ab64693	1:2500	Abcam (Cambridge, United Kingdom)
**MUM1**	MUM1p	1:400	Dako (Santa Clara, CA, United States)
**P53**	DO-7	1:400	Novocastra (Wetzlar, Germany)
**PAX5**	SP34	Ready to use	Roche (Basel, Switzerland)
**PD1**	NAT105	Ready to use	Roche (Basel, Switzerland)
**PD-L1**	28-8	1:100	Abcam (Cambridge, United Kingdom)
**pSTAT1**	EPR 3146	1:1500	Abcam (Cambridge, United Kingdom)
**TIA1**	TIA-1	1:50	Zytomed/Biocare (Berlin, Germany)

SupplementaryTable S2 *Detailed characteristics of the total collective*

Case #	Age (years)	Sex	Diagnosis	Subtype	EBV	Tx	Months Tx to PTLD	Follow-up
1	9	m	Non-destructive PTLD	FFH	+	HCT	22	Alive
2	36	m	Polymorphic PTLD		+	HCT	5	n/a
3	52	m	Polymorphic PTLD		+	HCT	2	Alive
4	28	m	Polymorphic PTLD		+	HCT	2	Dead
5	52	m	Polymorphic PTLD		+	HCT	4	Alive
6	17	m	Polymorphic PTLD		+	HCT	2	Dead
7	15	m	Polymorphic PTLD		-	HCT	3	Alive
8	52	m	Polymorphic PTLD		+	HCT	19	Alive
9	46	m	Monomorphic PTLD	DLBCL	+	HCT	23	Alive
10	28	m	Monomorphic PTLD	PBL	-	HCT	75	Alive
11	29	m	Monomorphic PTLD	PBL	+	HCT	2	Dead
12	19	f	Classic Hodgkin lymphoma PTLD		+	HCT	228	Alive
13	32	m	Non-destructive PTLD	PH	+	Liver	201	Alive
14	20	m	Non-destructive PTLD	FFH	+	Liver	12	Alive
15	66	m	Polymorphic PTLD		+	Liver	6	Alive
16	4	f	Monomorphic PTLD	DLBCL	+	Intestine	8	Alive
17	13	m	Monomorphic PTLD	BL	+	Liver	36	Alive
18	10	m	Monomorphic PTLD	DLBCL	-	Kidney	115	Alive
19	36	m	Monomorphic PTLD	DLBCL	-	Kidney	86	Alive
20	21	m	Monomorphic PTLD	DLBCL	-	Kidney	217	Alive
21	40	f	Monomorphic PTLD	PBL	+	Kidney	n/a	n/a
22	11	m	Monomorphic PTLD	DLBCL	-	Liver	84	Alive
23	17	f	Non-destructive PTLD	IM	+	HCT	2	Dead
24	74	m	Non-destructive PTLD	PH	-	HCT	23	Dead
25	38	m	Polymorphic PTLD		+	HCT	11	n/a
26	9	f	Polymorphic PTLD		+	HCT	13	Alive
27	25	f	Polymorphic PTLD		+	HCT	2	Alive
28	41	m	Polymorphic PTLD		+	HCT	1	Dead
29	15	f	Polymorphic PTLD		+	HCT	3	Alive
30	39	f	Polymorphic PTLD		+	HCT	1	Alive
31	60	f	Polymorphic PTLD		+	HCT	5	Dead
32	5	m	Polymorphic PTLD		+	HCT	4	Dead
33	26	f	Monomorphic PTLD	DLBCL	+	HCT	3	Dead
34	50	f	Monomorphic PTLD	Grey zone lymphoma*	-	HCT	48	Alive
35	41	m	Monomorphic PTLD	DLBCL	-	HCT	155	Dead
36	42	f	Monomorphic PTLD	DLBCL	+	HCT	2	Dead
37	5	f	Non-destructive PTLD	PH	+	Kidney	28	Alive
38	1	m	Polymorphic PTLD		+	Liver	9	Alive
39	60	m	Polymorphic PTLD		+	Multivisceral	3	Dead
40	13	m	Polymorphic PTLD		+	Heart	116	Alive
41	71	m	Polymorphic PTLD		+	Liver	n/a	n/a
42	12	m	Polymorphic PTLD		+	Intestine	46	Alive
43	60	m	Polymorphic PTLD		+	Liver	9	Dead
44	65	m	Polymorphic PTLD		+	Kidney	435	Dead
45	66	m	Monomorphic PTLD	DLBCL	-	Liver	19	Dead
46	9	m	Monomorphic PTLD	DLBCL	-	Heart	105	Alive
47	62	m	Monomorphic PTLD	ALCL	-	Lung	148	n/a
48	69	m	Monomorphic PTLD	HGBL with *MYC*- and *BCL2*-rearrangement	-	Liver	48	Alive

* B-cell lymphoma, unclassifiable, with features intermediate between DLBCL and Burkitt lymphoma {Jaffe ES, 2017 #95}.

PTLD = Post-transplant lymphoproliferative disorder
Tx = Transplantation
HCT = Hematopoietic stem cell transplantation
EBV = Epstein-Barr virus
m = male
f = female
n/a = not available

PH = Plasmacytic hyperplasia
IM = Infectious mononucleosis
FFH = Florid follicular hyperplasia
DLBCL = Diffuse large B-cell lymphoma
PBL = Plasmablastic lymphoma
HGBL = High grade B-cell lymphoma
ALCL = Anaplastic large cell lymphoma
BL = Burkitt lymphoma

Supplementary Table S3 *Macrophages polarization of the 22 TMA cases*

**Case #**	**Polarization**
1	M1
2	Intermediate
3	Intermediate
4	M1
5	M1
6	M1
7	M1
8	M1
9	M1
10	M1
11	M1
12	M1
13	M2
14	M1
15	M2
16	M1
17	M1
18	M2
19	M1
20	M1
21	M2
22	M1

M1-polarization: ratio of CD163/pStat1-positive cells to CD163_cMaf-positive cells > 1.5.
M2-polarization: ratio of CD163/cMaf-positive to CD163/pStat1-positive cells > 1.5.
Intermediate: If neither ratio was > 1.5

Supplementary Fig S1 Basic data of the total collective

Comparison of the total collective and the subgroup analyzed on TMA shows representative results regarding patient characteristics and PTLD-associated features of the TMA-subgroup.

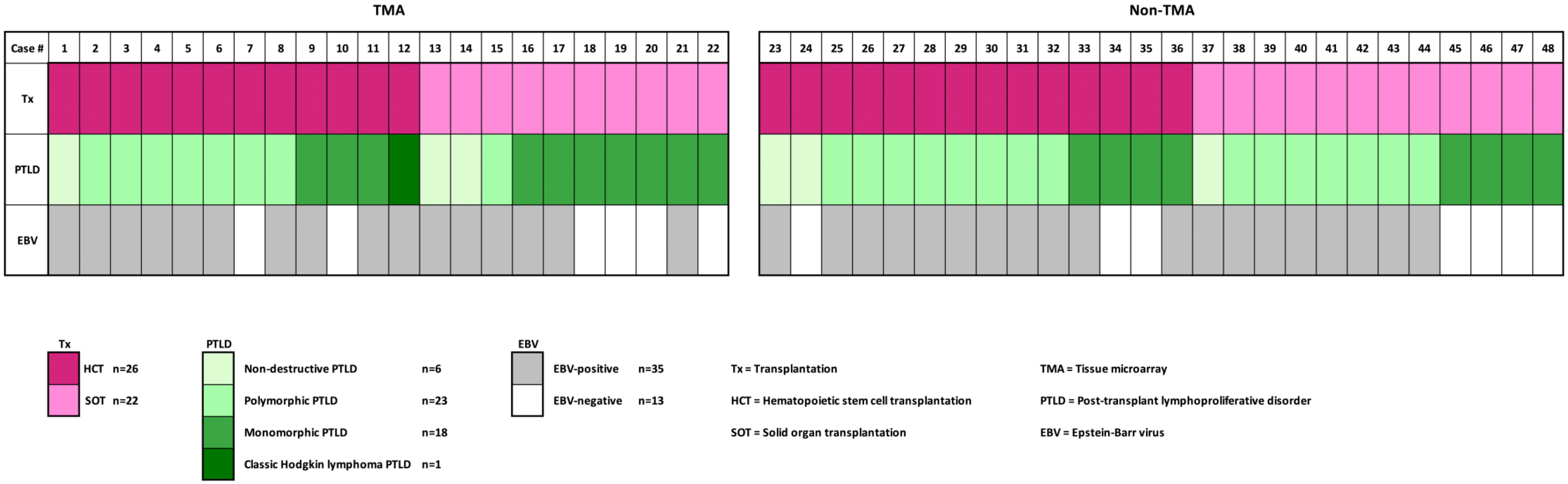

Supplementary Fig S2 Morphological and immunophenotypical features of a polymorphic EBV negative PTLD (case #7) Low power magnification highlights the effaced architecture (a, hematoxylin-eosin (H&E) stain, original magnification x50; insert EBER in-situ hybridization, x100). The polymorphic lymphoid proliferation comprises mature lymphocytes, blasts and plasma cells intermingled with scattered macrophages (b, H&E stain, x400). The immunophenotype of the lymphoid infiltrate shows a heterogenous staining for PAX5 (c) and positivity for CD79a (d). CD3 staining reveals a prominent reactive T cell population (e) (C-E, x200).

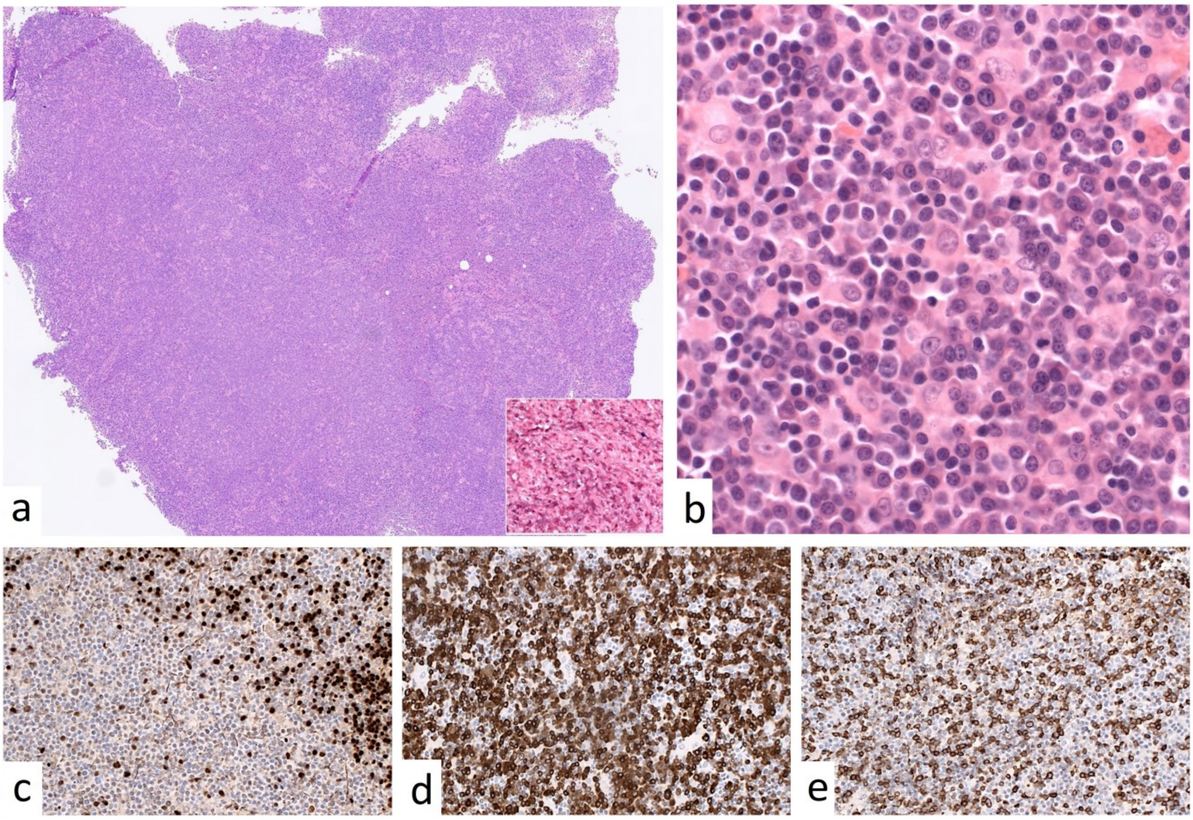

Supplement: Supplementary file 1 — (PDF 1680 kb) [file 428_2020_2985_MOESM1_ESM.pdf]
